# The geography of Medicare's hospital value‐based purchasing in relation to market demographics

**DOI:** 10.1111/1475-6773.14141

**Published:** 2023-02-22

**Authors:** Colleen C. McLaughlin, Francis P. Boscoe

**Affiliations:** ^1^ Department of Population Health Sciences Albany College of Pharmacy and Health Sciences Albany New York USA; ^2^ Pumphandle, LLC Camden Maine USA

**Keywords:** hospitals, patient satisfaction, pay for performance, quality of healthcare, spatial analysis, systemic racism

## Abstract

**Objective:**

To illustrate the association between the sociodemographic characteristics of hospital markets and the geographic patterns of Medicare hospital value‐based purchasing (HVBP) scores.

**Data Sources and Study Setting:**

This is a secondary analysis of United States hospitals with a HVBP Total Performance Score (TPS) for 2019 in the Centers for Medicare and Medicaid Services (CMS) Hospital Compare database (4/2021 release) and American Community Survey (ACS) data for 2015–2019.

**Study Design:**

This is a cross‐sectional study using spatial multivariable autoregressive models with HVBP TPS and component domain scores as dependent variables and hospital market demographics as the independent variables.

**Data Collection/Extraction Methods:**

We calculated hospital market demographics using ZIP code level data from the ACS, weighted the 2019 CMS inpatient Hospital Service Area file.

**Principal Findings:**

Spatial autoregressive models using eight nearest neighbors with diversity index, race and ethnicity distribution, families in poverty, unemployment, and lack of health insurance among residents ages 19–64 years provided the best model fit. Diversity index had the highest statistically significant contribution to lower TPS (*ß* = −12.79, *p* < 0.0001), followed by the percent of the population coded to “non‐Hispanic, some other race” (*ß* = −2.59, *p* < 0.0023), and the percent of families in poverty (*ß* = −0.26, *p* < 0.0001). Percent of the population was non‐Hispanic American Indian/Alaskan Native (*ß* = 0.35, *p* < 0.0001) and percent non‐Hispanic Asian (*ß* = 0.12, *p* < 0.02071) were associated with higher TPS. Lower predicted TPS was observed in large urban cities throughout the US as well as in states throughout the Southeastern US. Similar geographic patterns were observed for the predicted Patient Safety, Person and Community Engagement, and Efficiency and Cost Reduction domain scores but are not for predicted Clinical Outcomes scores.

**Conclusions:**

The lower predicted scores seen in cities and in the Southeastern region potentially reflect an inherent—that is, structural—association between market sociodemographics and HVBP scores.

**What is known on this topic**

- Medicare's hospital value‐based purchasing program scores have been shown to be lower for safety‐net hospitals, resulting in reduced Medicare reimbursement.
- There has been a call to adjust the scores for the socioeconomic status of patients seen by each hospital, but regulators have resisted holding these hospitals to a lower standard.
- The local and regional geographic impact of these policies across the United States has not been explored.

**What this study adds**

- We created maps showing how the hospital scores were potentially affected by the sociodemographic characteristics of their market areas.
- Hospitals in the Southeastern states and larger cities with racial and ethnic diversity had lower predicted scores, potentially representing an increased risk of lower hospital value‐based purchasing program scores.
- Patient experience, patient safety, and hospitalization related costs contributed to the lower predicted scores. There was less evidence of regional geographic patterns in clinical quality.

## 1 INTRODUCTION

The Centers for Medicare and Medicaid Services (CMS) implemented hospital value‐based purchasing (HVBP) for acute care hospitals starting with 2012 discharges as part of the Affordable Care Act.^1^ Through this program, Medicare withholds 2% of participating hospitals' base operating Medicare severity diagnosis‐related group (MS‐DRG) payment amount, which is then redistributed across all participating hospitals based on relative performance and demonstrated improvement. In 2019, $1.9B was redistributed among almost 2800 hospitals, with 44% of hospitals having a net loss in Medicare income and 5% losing more than $350K.^2^, ^3^

Numerous commentators have noted that as the US payer system moves toward pay‐for‐performance/value‐based purchasing, there is a risk of increased penalization of safety‐net hospitals.^4^, ^5^, ^6^, ^7^, ^8^, ^9^, ^10^, ^11^, ^12^, ^13^ The “consistent, negative, and significant effect”^13^ of safety‐net status on HVBP scores implies an association with factors that are outside hospitals' ability to modify, such as market characteristics, government payer distribution, and ownership structure.^8^ Lower access to high‐quality health care is a subset of the inequalities faced by communities of concentrated poverty, and by extension, communities where racial or ethnic minority groups are overrepresented.^12^, ^14^, ^15^, ^16^ At the patient and community level, therefore, there is the potential for HVBP incentive payments to reinforce structural racism.

While the association between safety‐net hospital status and lower overall performance on HVBP has been explored in a growing body of literature, the extent and patterns of the impact of the racial and socioeconomic make‐up of patient populations on HVBP scores across the US have not been explored from a geospatial perspective. Our objective is to apply spatial data analysis to map the association between hospital markets and HVBP scores across the US, illustrating both local detail and broader regional trends. Our work fits into the public health tradition of disease mapping wherein the local nature of spatial patterning of data is explicitly used to improve estimation.^17^ Our use of spatial models, increased specificity in defining hospital market areas, and national scope represent enhancements to what is known about structural racism and HVBP.

## 2 METHODS

### 2.1 Study population and outcome measures

This is a cross‐sectional observational study of all non‐federal general medical/surgical hospitals in the United States (US) receiving scores for 2019 discharges in the CMS HVBP program, as of the April 1, 2021 release (*n* = 2673).^3^ We used the HVBP Total Performance Score (TPS) and its component domain scores as our outcome measures. The TPS is the sum of four domain scores: Person Score from Hospital Consumer Assessment of Healthcare Providers and Systems (HCAHPS) survey of patient and family experience; Safety Score based on selected hospital acquired infections; Clinical Outcomes Score based on 30‐day risk‐standardized mortality rates for three conditions and one complication measure; and Efficiency and Cost Reduction Score derived from Medicare spending per beneficiary. We examined 2019 HVBP scores to avoid potential confounding by COVID‐19; the 2019 Clinical Outcomes domain was based on patient discharged from July 2016 to June 2019 (30‐day mortality measures) and April 2016 to March 2019 (Hip/Knee replacement complication measure).

### 2.2 Independent variables

We defined flexible hospital markets based on ZIP code level data using CMS's Market Service Area file for hospitals derived from Medicare fee‐for‐service recipients.^18^ Hospital market was estimated from the 2019 CMS inpatient Hospital Service Area File.^18^ This file provides a count of services rendered to fee‐for‐service Medicare beneficiaries by facility and by the beneficiary's mailing ZIP code, excluding hospital/ZIP codes pairs with 10 or fewer discharges. Population demographics for 5‐digit Zip Code Tabulation Areas (ZCTA) were obtained from the American Community Survey (ACS) 5‐year estimates for 2015–2019.^19^ We assumed a one‐to‐one correlation between ZCTA and ZIP code. ZIP codes on the market file but not the ACS file (9%) were excluded. These most likely represent PO Box only ZIP codes. Hospital market values were estimated by weighting the ZCTA estimates from the ACS by the percent of the total hospital market in each ZIP code/ZCTA as follows:

$$\text{Hospital market}\left(i\right)=\sum_{j=1}^{n}\frac{\text{Hospital cases}\in \text{ZCTA}\left(\mathrm{ji}\right)}{\text{Total hospital cases}\left(i\right)}*\text{ZCTA estimate}\left(j\right).$$

Market characteristics obtained from ACS included weighted percentages of hospital markets for race/ethnicity, racial/ethnic diversity, high school graduates, unemployment, foreign born, families living below the federal poverty level, and lack of health care insurance among persons aged 19–64. Race and ethnicity as collected for the ACS are self‐reported and categorized based on the 1997 Office of Management and Budget standards.^20^ We used all categories tabulated in the ACS public use files: mutually exclusive Hispanic, Black, White, American Indian/Alaska Native (AIAN), Asian, Native Hawaiian or other Pacific Islander (PI), some other race, and two or more races. Racial/ethnic diversity was measured using the US Census Diversity Index, which is a measure of a probability of two people chosen at random from the population being of different race/ethnic groups.^21^ Other ACS variables beyond these, such as non‐English languages spoken at home, food stamp recipients, and public insurance, were considered and excluded due to high collinearity (Pearson correlation coefficient greater than 0.75) with other variables.

Institutional Review Board review was not necessary since this study did not meet the definition of human subjects research. All data used are publicly available.

### 2.3 Statistical analysis

We applied spatial autoregressive (SAR) models to each dependent variable to estimate the predicted scores based on individual hospital's independent variables and the observed scores of neighboring hospitals. These spatial models account for the spatial dependence between observations (that is, observations in one location tend to be similar to observations in other locations). These models are more conservative and less biased than ordinary least squares (OLSs) regression models, particularly given the spatial dependency (i.e., overlap) in hospital markets.

A formal model selection process was used to determine the appropriate form of the multivariable spatial regression. A nearest‐neighbor (*k*) matrix for each hospital was created for each hospital for *k* = 6–10 neighbors per hospital. Imputation was used to assign missing values for the HVBP domain scores, which were missing for a small number of hospitals, which allowed the same neighbor matrix to be used for all models.

We used a backward elimination process to select the independent variables used in the analysis. We split the data into training (70%) and test (30%) data sets and fit OLS regression models to the training data with TPS as the outcome measure, beginning with the full set of variables listed above and removing the variable with the lowest *t*‐statistic (highest *p*‐value). Each candidate model was then applied to the test data, and the final model was selected that had the most candidate variables without loss‐of‐fit, for example, where the mean squared error leveled off. The variables in this final model were then used to fit a SAR model on the entire data set for TPS. We compared the SAR model using *k* = 6–10, and the *k* with the best model fit using the Akaike information criterion was used for all subsequent models. For consistency, the same model was used for the HVBP component scores.

Because the predictors in SAR models include both the modeled variables and the dependent variables for the k‐nearest neighbors, the interpretation of the estimated coefficients is not as straightforward as for OLSs, although the direction and magnitude of the coefficients retain their interpretive properties.^22^ The estimated coefficients represent the direct effect of the independent variables on the outcome and are constant across hospitals. Predicted HVBP scores for individual hospitals are a combination of these direct effects and indirect effects based on the HVBP scores of the hospital's *k*‐neighbors.

We created dot maps of the observed and predicted HVBP scores to allow comparison of regional patterns. Maps of the predicted values from SAR models show the influence of smoothing the dependent variables based on the local (*k*‐neighbor) patterns of the independent variables.^23^ In other words, spatial autoregression reduces the effect of factors not in the spatial model.

PROC SPATIALREG in SAS v9.4 was used to perform the spatial analysis. Given the computational complexity of fitting an SAR model to large datasets, we used the Chebyshev approximation to calculate the eigenvalues. The maps were produced in R using the usmap and ggplot2 packages.

## 3 RESULTS

Of the 3158 acute care hospitals in the 2019 Hospital Care Compare dataset from 49 US states, 2673 (85%) had HVBP TPS and data on ZIP codes of their Medicare fee‐for‐service patients on the CMS service area file.

### 3.1 Model selection

The selected model included 11 variables (Table 1). In the SAR models, the number of nearest neighbors per hospital that produced the best model fit was 8 although there was little difference in model fit between 6 and 10 neighbors.

**TABLE 1:** The association between hospital value based purchasing scores^a^ and hospital market sociodemographic measures^b^ using spatial autoregression, United States Acute Care Hospitals,^c^ 2019.

	Total performance score		Clinical outcomes		Safety		Person and community engagement		Efficiency and cost reduction	
	Estimate^d^	*p*‐value	Estimate	*p*‐value	Estimate	*p*‐value	Estimate	*p*‐value	Estimate	*p*‐value
Intercept	29.90	<0.0001	8.72	<0.0001	9.61	<0.0001	9.58	<0.0001	6.21	<0.0001
Diversity index	−12.79	<0.0001	−1.74	0.0667	−4.08	0.0003	−3.06	0.0045	−7.85	<0.0001
Percent Hispanic	0.03	0.1081	0.04	<0.0001	0.02	0.0969	−0.04	0.0001	0.02	0.1338
Percent Black^e^	0.04	0.1187	0.03	0.0154	0.01	0.4741	−0.01	0.4749	0.02	0.2486
Percent AIAN	0.35	<0.0001	0.07	0.0006	0.05	0.0531	0.05	0.0413	0.28	<0.0001
Percent Asian	0.12	0.0207	0.08	<0.0001	0.03	0.2552	−0.02	0.4469	0.02	0.5208
Percent PI	0.19	0.6904	−0.32	0.1006	0.03	0.9082	0.36	0.1047	0.34	0.2472
Percent some other race	−2.59	0.0023	0.19	0.5945	−0.81	0.0529	−1.79	<0.0001	−1.12	0.0372
Percent two or more races	0.40	0.0543	0.05	0.5391	0.12	0.2321	−0.11	0.2511	0.21	0.1055
Percent of families below poverty line	−0.26	0.0013	−0.07	0.0429	−0.08	0.0494	0.00	0.9067	−0.13	0.0113
Unemployment rate	−0.18	0.2919	−0.16	0.0235	0.07	0.4325	−0.25	0.0024	0.17	0.1131
Percent residents ages 19–64 years without health insurance	0.01	0.7958	−0.03	0.1424	0.08	0.0011	0.04	0.1332	−0.04	0.2648
Rho	0.32	<0.0001	0.35	<0.0001	0.13	0.001	0.31	<0.0001	0.46	<0.0001
Sigma‐squared	116.32	<0.0001	19.77	<0.0001	28.03	<0.0001	25.89	<0.0001	46.54	<0.0001

Abbreviations: AIAN, American Indian/Alaska Native; PI, Pacific Islander.

^a^ Centers for Medicare & Medicaid Services, Hospital Care Compare April 2021 release.

^b^ American Community Survey 2015–2019.

^c^ Includes all acute care hospitals in the United States receiving Hospital Value‐Based Purchasing Total Performance Score for 2019; includes District of Columbia; excludes Maryland and US territories.

^d^ Estimated coefficients represent the direct effect of the covariate on the outcome and are constant across all observations.

^e^ All listed racial groups are from the American Community Survey non‐Hispanic or Latino population estimates.

The autoregressive regression coefficient, rho, was statistically significant in all models, indicating all the HVBP variables examined displayed spatial dependence. All models also had statistically significant residual variance (measured by sigma‐squared), which can be due to outliers in the predicted HVBP scores or to the presence of regional patterns in addition to the modeled local patterns.

Of the included variables, racial/ethnic diversity had the strongest association by a large margin, although it did not reach statistical significance in the model for clinical outcomes. Once adjusted for diversity, the other race and ethnicity categories tend to have positive, albeit weaker, associations with the HVBP outcomes, indicating hospitals serving communities with higher concentrations of residents of specific racial or ethnic groups tended to perform slightly better. For example, the five hospitals with markets that were 75% or more non‐Hispanic AIAN had a low diversity index (mean 0.21, US hospitals overall mean 0.42), but a high observed TPS (mean 63, US hospitals overall mean 34). Three of these facilities were Indian Health Service hospitals, and the other two were tribal hospitals.

### 3.2 Model results

**FIGURE 1:**
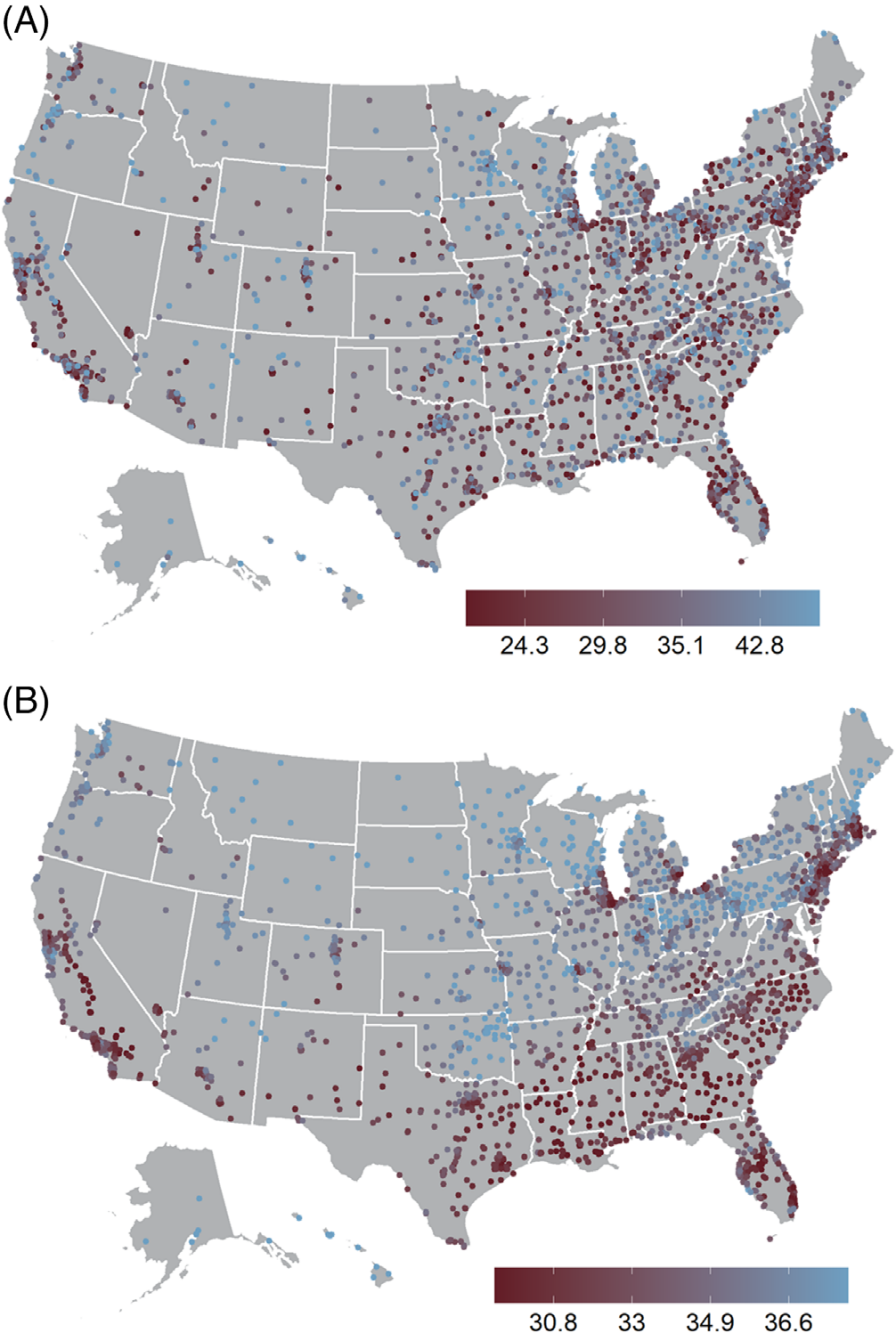
Observed and predicted hospital value‐based purchasing total performance scores, 2019, United States Acute Care Hospitals,^1^ Centers for Medicare and Medicaid Services Hospital Care Compare April 2021 release. (A) Observed Total Performance Scores, (B) Predicted Total Performance Score.^2^ 1. Includes all acute care hospitals in the United States receiving Hospital Value‐Based Purchasing Total Performance Score for 2019; includes District of Columbia; excludes Maryland and US territories. 2. Predicted scores based on spatial autoregressive models of estimated hospital market sociodemographic characteristics (racial/ethnic diversity; the percentage of population that is, respectively, Hispanic, Black, American Indian/Alaska Native, Asian, Native Hawaiian or other Pacific Islander, some other race, and two or more races; the percent of families living below the federal poverty level; percent of individuals age 19–64 years without health insurance; and unemployment rate.) These predicted values represent smoothed estimates based on the market sociodemographic characteristics of the individual hospital and hospital value‐based purchasing scores of the hospital's eight nearest neighbors. [Color figure can be viewed at wileyonlinelibrary.com]

There is clear spatial patterning to the predicted TPS that is not as evident in the observed scores (Figure 1). Regionally, the South Atlantic, East South Central, and West South Central regions have the most evidently lower predicted TPS scores based on the modeled hospital market effects, hereafter referred to as “sociodemographic composition.” Pockets of lower predicted TPS scores were also observed in the large urban areas across the entire country, including the northeast urban corridor (Boston–New York City–Philadelphia–Washington DC), cities in the Great Lakes area (Cleveland, Detroit, Chicago), and both northern California (Los Angeles) and southern California (San Francisco).

**FIGURE 2:**
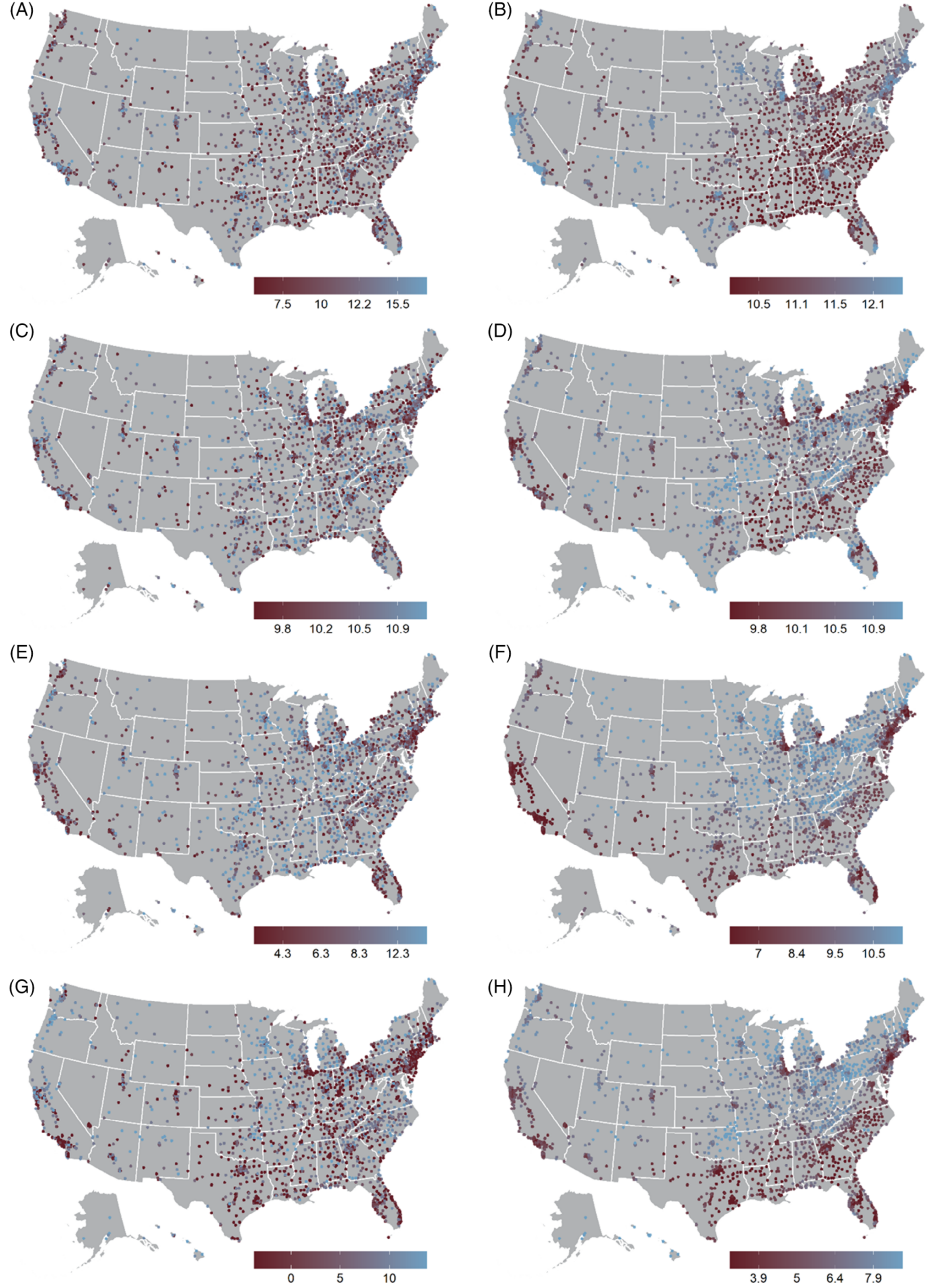
Observed and Predicted Hospital Value‐Based Purchasing Domain Scores, 2019, United States Acute Care Hospitals,^1^ Centers for Medicare and Medicaid Services Hospital Care Compare April 2021 release. (A) Observed Clinical Outcomes Score, (B) Predicted Clinical Outcomes Score,^2^ (C) Observed Safety Score, (D) Predicted Safety Score,^2^ (E) Observed Person and Community Engagement Score, (F) Predicted Person and Community Engagement Score,^2^ (G) Efficiency and Cost Reduction Score, (H) Predicted Efficiency and Cost Reduction Score.^2^ 1. Includes all acute care hospitals in the United States receiving Hospital Value‐Based Purchasing Total Performance Score for 2019; includes District of Columbia; excludes Maryland and US territories. 2 Predicted scores based on spatial autoregressive models of estimated hospital market sociodemographic characteristics (racial/ethnic diversity; the percentage of population that is, respectively, Hispanic, Black, American Indian/Alaska Native, Asian, Native Hawaiian or other Pacific Islander, some other race, and two or more races; the percent of families living below the federal poverty level; percent of individuals age 19–64 years without health insurance; and unemployment rate.) These predicted values represent smoothed estimates based on the market sociodemographic characteristics of the individual hospital and hospital value‐based purchasing scores of the hospital's eight nearest neighbors. 3. Missing values were imputed as follows: Efficiency and Cost Reduction Score *n* = 1, 0.0%; Person and Community Engagement Score *n* = 6, 0.2%; Clinical Outcomes Score *n* = 44, 1.6%; and Safety Score *n* = 507, 19.3%. [Color figure can be viewed at wileyonlinelibrary.com]

Similar spatial patterning was observed for the predicted Person and Community Engagement Score, predicted Safety Score, and predicted Efficiency and Cost Reduction Scores (Figure 2). While the maps of the predicted outcomes are visually similar, differences can be seen in specific areas, such as in Oklahoma, where hospitals were ranked lower for predicted Person and Community Engagement relative to their rankings for predicted TPSs (Figure 2). The predicted Clinical Outcome Scores did not demonstrate clear spatial patterning with respect to the southern regions of the US, reflecting the smaller *ß* coefficients for the sociodemographic variables in the models that generated these estimates. On the other hand, many urban areas had higher predicted Clinical Outcomes Scores.

## 4 DISCUSSION

We used spatial autoregression to predict HVBP scores based on the sociodemographic composition of each hospital's market. Our principal result is that accounting for the sociodemographic composition of hospital markets on HVBP TPS results in increased risk of low scores in large urban cities as well as in the Southeastern United States. When the individual domains of the HVBP scores are examined, the Person and Community Engagement, Safety, and Efficiency and Cost Reduction domains reflect the same regional and urban patterns. Clinical outcomes, on the other hand, show clustering of higher predicted scores in large urban areas. Our results complement the recent report by Khullar et al., who reported that the US Census Bureau’ South Atlantic and East South Central regions and counties with a higher proportion Black residents had a disproportionate proportion of hospitals that were low‐performing across all three CMS value‐based programs.^24^ Our findings add to Khullar's by demonstrating that the high proportion of low‐performing hospitals in the Southeast could be explained by the sociodemographics of the region, rather than inherently less reliable health care delivery independent of sociodemographics. Our finding that the Clinical Outcomes domain did not exhibit the same geographic pattern as the other HVBP domains supports this hypothesis. To our knowledge, this is the first published work on the geographic pattern of the association between HVBP and hospital market demographics. The statistically significant autoregressive regression coefficient in all models indicates that our results may be conservative relative to non‐spatial models, which run the risk of type I error when spatial dependency is present.^25^

Of all the sociodemographic variables examined, the US Census Bureau's diversity index had the strongest association in the SAR models. This reflects the idea that areas with higher percentages of racial and ethnic diversity tended to have lower‐performing hospitals, regardless of what specific racial or ethnic minority group predominated. While many studies of inequality in access to quality health care concentrate on the lower availability of health care, there is a growing body of literature reporting on the association between hospital quality and minority‐service hospital status across multiple health states and conditions.^4^, ^5^, ^8^, ^10^, ^13^, ^26^, ^27^, ^28^, ^29^, ^30^, ^31^, ^32^, ^33^, ^34^, ^35^, ^36^, ^37^, ^38^, ^39^, ^40^, ^41^, ^42^, ^43^, ^44^, ^45^, ^46^, ^47^, ^48^, ^49^ Minority‐serving hospitals have also been shown to have lower scores for reported patient experience, despite findings that minority patients report better experiences overall.^12^, ^50^, ^51^ A consistent finding has been that disparities in patient‐level health risks alone do not explain variation in quality between hospitals. In other words, the quality disparity associated with minority‐serving hospitals is likely due to systemic factors.^46^

Multiple commentators have suggested that pay‐for‐performance measures and publicly reported star ratings should be adjusted for neighborhood or patient demographic characteristics.^9^, ^52^, ^53^, ^54^ CMS makes two arguments against the adjustment of HVBP measures for race and ethnicity or socioeconomic status.^1^ First, it would be akin to holding hospitals with more diverse patient populations to a different standard. Second, it would obscure important differences. We believe the arguments could be more nuanced. In particular, since MSPB includes both costs associated with both the inpatient stay and 30 days post‐discharge, adjusting the Efficiency and Cost Reduction scores for the sociodemographic status of the hospital market could be seen as leveling the playing field for populations that may be medically underserved. Care would be needed to assure that such modifications do not have the unintended consequence of reflecting the lower quality and safety of the index inpatient episode.^55^ In contrast, the argument for adjusting the HVBP Person and Community Engagement scores for socioeconomic risk is also not clear‐cut. Safety‐net or minority‐serving hospital status should not be synonymous with lower patient satisfaction.^7^, ^12^, ^50^, ^51^, ^56^, ^57^ Our analysis demonstrates that the Clinical Outcomes domain does not share the sociodemographic‐based patterning of the other HVBP domains, suggesting that adjustment for sociodemographic risk would be unnecessary.

As a cross‐sectional ecological study, we need to be careful not to over‐interpret these findings. Translating these findings into a causal relationship between HVBP incentive payments and poorer quality care received by individuals, particularly in specific demographic groups or regions, is beyond the scope of this study.^58^ We chose to highlight the spatial patterning of the association with hospital market to add to the understanding of potential structural racism in CMS's HVBP program. There are multiple other hospital characteristics, such as size, ownership structure, and teaching status, that also influence hospital quality and have spatial dependence.

## 5 LIMITATIONS

Wider understanding of potential structural racism in access to higher quality care is hindered by the lack of nationwide data on quality‐of‐care data stratified by patient demographics at the hospital or community level. Researchers interested in the demographic geography of the hospital service areas have generally been limited to defining service areas based on the physical location of the hospital, ranging in scale from block group or census tract^7^, ^52^, ^59^, ^60^ to county.^15^, ^60^ Smaller‐scale geographies will underestimate the market in rural areas, while larger scales will overestimate the market in cities, particularly those with multiple hospitals with overlapping service areas. Our use of the Medicare service area file allowed us to define markets that are flexible across the urban/rural continuum and are weighted based on the actual patterns of hospital/ZIP code patient flow.

Use of the Medicare fee‐for‐service population may misestimate hospital service areas in areas where (1) there is heterogeneity in market share by patient age and (2) there is local geographic heterogeneity in the adoption of Medicare Advantage adoption. Our calculation of market demographics also assumes that hospital choice by residents of a specific ZIP code does not vary by the demographic factors examined. Previous studies have shown this assumption is likely violated; White patients are more likely to travel further for care.^61^, ^62^, ^63^, ^64^, ^65^ In the case of a hospital serving a ZIP code from which White patients differentially travel to other hospitals, we are underestimating the percent of minority patients that the hospital actually serves. Regardless, our estimation method provides increased specificity in market area definition compared to other methods available for use at the national scale.

## 6 CONCLUSION

Our findings highlight the potential for structural racism in the HVBP program, with lower predicted scores for hospitals in the Southeastern states and larger cities in the US that have higher levels of racial and ethnic diversity. In particular, we show a consistent finding for a spatial relationship between market sociodemographic composition and the HVBP Person and Community Engagement and Efficiency and Cost Reduction domains, both of which have been shown in previous studies to be associated with hospital patient mix. We also show this spatial patterning for the safety domain, which has not been as well documented. This exploratory study complements existing understanding of place‐based structural racism on value‐based payment metrics and further highlights the need for re‐evaluation of policy regarding adjustment of incentive payments to reduce disparities among hospitals that serve minority populations.
